# Dietary intake and risk of rheumatoid arthritis—a cross section multicenter study

**DOI:** 10.1007/s10067-016-3383-x

**Published:** 2016-08-23

**Authors:** Jing He, Yu Wang, Min Feng, Xia Zhang, Yue-Bo Jin, Xue Li, Lin-Chong Su, Shuang Liu, Ai-Xue Wang, Xiao-Mei Chen, Li-Jun Wu, Xiao-Xia Yu, Ning Xu, Xiang-Yuan Liu, Hui-Ming Yan, Yong-Fu Wang, Bin Jia, Jun-Fang Li, Jie-Mei Tao, Feng-Xiao Zhang, Ping Yu, Liu-Fu Cui, Jing Yang, Zhen-Bin Li, Jian-Li Xie, Ping Wei, Wen-Wen Sun, Lu Gong, Yong-Jing Cheng, Ci-Bo Huang, Xiao-Yuan Wang, Yi Wang, Hui-Fang Guo, Hong-Tao Jin, Xia Liu, Guo-Chun Wang, Yan-Hua Wang, Lan He, Yi Zhao, Xiao-Xia Li, Yan Zhang, Jian-Ping Guo, Zhan-Guo Li

**Affiliations:** 1 Department of Rheumatology and Immunology, Peking University People’s Hospital, 11 Xizhimen South Street, Beijing, 100044 China; 2 School of Statistics and The Center for Applied Statistics, Renmin University of China, Beijing, 100872 China; 3 Department of Rheumatology and Immunology, University Hospital of Hubei University for Nationalities, Enshi, 445000 China; 4 Department of Rheumatology and Immunology, People’s Hospital of Xinjiang Uygur Autonomous Region, Urumqi, 830001 China; 5 Department of Rheumatology and Immunology, Cangzhou Hospital of Integrated Traditional Chinese and Western Medicine of Hebei Province, Cangzhou, 061001 China; 6 Department of Rheumatology and Immunology, Peking University Third Hospital, Beijing, 100191 China; 7 Department of Rheumatology and Immunology, The First Affiliated Hospital of Baotou Medical College, Baotou, 014010 China; 8 Department of Rheumatology and Immunology, Hebei Province Central Hospital of Handan City, Handan, 056001 China; 9 Department of Rheumatology and Immunology, Hebei General Hospital, Shijiazhuang, 050051 China; 10 Department of Rheumatology and Immunology, Kailuan General Hospital, Tangshan, 063000 China; 11 Department of Rheumatology and Immunology, Bethune International Peace Hospital, Shijiazhuang, 050082 China; 12 Department of Rheumatology and Immunology, The Third Affiliated Hospital of Hebei Medical University, Shijiazhuang, 050052 China; 13 Department of Rheumatology and Clinical Immunology, General Hospital of Tianjin Medical University, Tianjin, 300052 China; 14 Department of Rheumatology and Immunology, Beijing Hospital, Beijing, 100005 China; 15 Department of Rheumatology and Immunology, Lanzhou University Second Hospital, Lanzhou, 730030 China; 16 Department of Rheumatology and Immunology, The Second Hospital of Hebei Medical University, Shijiazhuang, 050000 China; 17 Department of Rheumatology and Immunology, China-Japan Friendship Hospital, Beijing, 100029 China; 18 Department of Rheumatology and Immunology, The First Affiliated Hospital of Xi’an Jiaotong University, Xi’an, 710061 China; 19 Department of Rheumatology and Immunology, Beijing Xuanwu Hospital, Beijing, 100053 China; 20 Department of Rheumatology and Immunology, The 309th Hospital of Chinese People’s Liberation Army, Beijing, 100000 China

**Keywords:** Chinese population, Dietary factors, Disease susceptibility, Rheumatoid arthritis

## Abstract

Environmental factors play an important role in the development of rheumatoid arthritis (RA). Among these factors, smoking is generally considered to be an established risk factor for RA. Data regarding the impact of diet on risk of RA development is limited. This study assessed the impact of dietary patterns on RA susceptibility in Chinese populations. This was a large scale, case-control study composed of 968 patients with RA and 1037 matched healthy controls. Subjects were recruited from 18 teaching hospitals. Socio-demographic characteristics and dietary intakes 5 years prior to the onset of RA were reported by a self-administered questionnaire. Differences in quantity of consumption between cases and controls were analyzed by Student’s *t* test. Multiple logistic regression analysis was applied to identify independent dietary risk factor(s) responsible for RA susceptibility. Compared to healthy individuals, RA patients had decreased consumption of mushrooms (*P* = 0.000), beans (*P* = 0.006), citrus (*P* = 0.000), poultry (*P* = 0.000), fish (*P* = 0.000), edible viscera (*P* = 0.018), and dairy products (*P* = 0.005). Multivariate analyses revealed that several dietary items may have protective effects on RA development, such as mushrooms (aOR = 0.669; 95%CI = 0.518–0.864, *P* = 0.002), citrus fruits (aOR = 0.990; 95%CI = 0.981–0.999, *P* = 0.04), and dairy products (aOR = 0.921; 95%CI 0.867–0.977, *P* = 0.006). Several dietary factors had independent effects on RA susceptibility. Dietary interventions may reduce the risk of RA.

## Introduction

Rheumatoid arthritis (RA) is a chronic systemic inflammatory disease that leads to progressive joint erosion and destruction and affects 0.5–1 % of the population. RA is considered to be a complex disease that is triggered by both genetic and environmental factors, and the pathogenesis of RA has not yet been fully elucidated. Recent advances in high-throughput genome scanning have enabled the identification of many genetic risk factors that contribute to RA susceptibility. However, it has been estimated that the genetic factors account for only 60 % of the risk for RA susceptibility [1], while environmental and other non-genetic factors account for the remaining 40 %.

Among environmental factors, while only smoking is currently considered to be an established risk factor for RA [2, 3], other factors, such as diet, may contribute to RA susceptibility. Diet is an environmental factor that affects inflammation, antigen presentation, antioxidant defense mechanisms, allergies, and gut microbiota, and the exact impact of diet on RA risk remains uncertain. Several case-control studies have suggested that omega-3 fatty acids, which are present in fish, soybean, safflower, sunflower, and corn oils, may confer protection against RA [4, 5]. Alcohol may reduce the risk of RA in women [6]. While reactive oxygen species (ROS) are produced in rheumatoid joints [7], the roles of dietary factors such as antioxidants and vitamins in the pathogenesis of RA remain unclear. Vitamin C is both an intracellular and an extracellular scavenger of ROS, and in models of rat adjuvant arthritis supplementation with vitamin C contributed to improved arthritis scores, evidenced by a reduction in paw volume [8, 9]. However, there is little clinical evidence on the effects of antioxidants, such as vitamins E and C, on individuals with RA [10, 11]. A 2-year study on the effects of the Mediterranean diet on RA patients revealed that the consumption of cereals, vegetables, legumes, fruits, and olive oil decreased the risk of new onset of inflammatory polyarthritis [12].

While there is evidence on the effects of diet on RA pathogenesis in Western populations, there is little information to date concerning the impact of diet on the development of RA in Chinese populations. Here, we assess the impact of dietary factors on the development of RA in Chinese populations.

## Patients and methods

### Study population

**Fig. 1:**
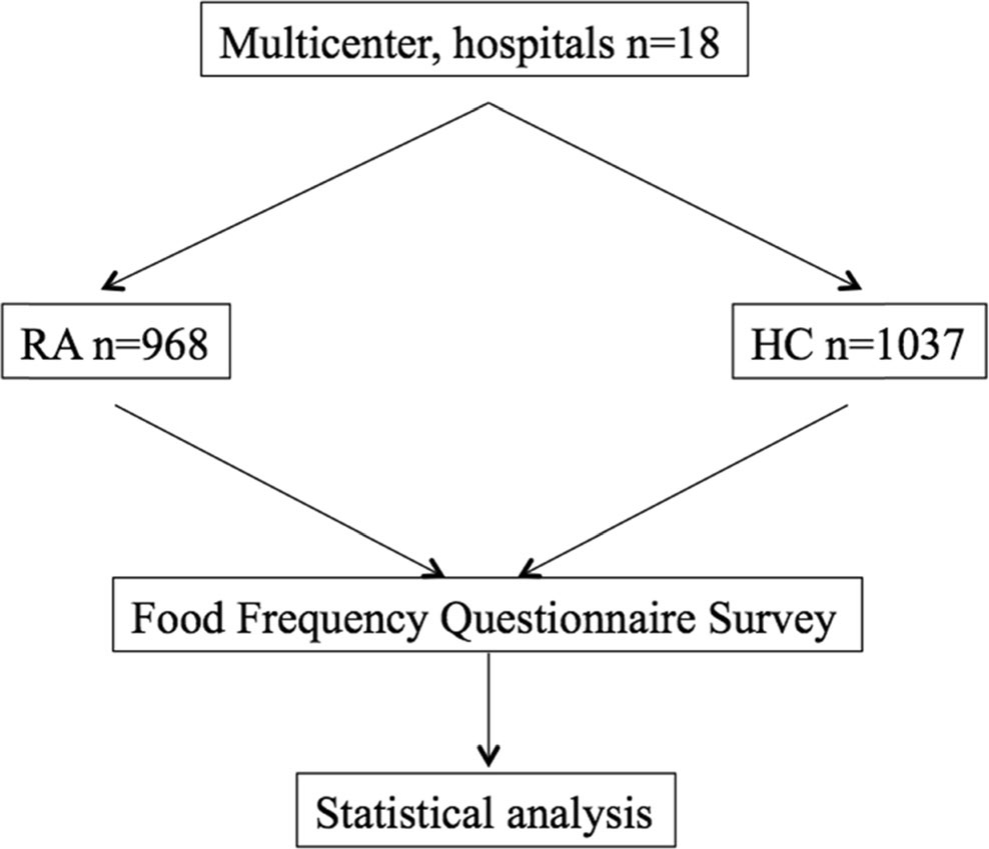
Patient flow chart. *RA* rheumatoid arthritis, *HC* healthy controls. A total of 968 RA patients and 1037 healthy controls were recruited from 18 hospitals

A total of 968 RA patients and 1037 ethnic and geographically matched healthy controls were recruited from 18 hospitals (Fig. 1). All RA patients fulfilled the American College of Rheumatology (ACR) criteria for RA [13]. Healthy controls were randomly selected from medical workers without any history of chronic diseases. A self-administered food frequency questionnaire (FFQ) was designed to measure socio-demographic characteristics and dietary intakes. Patients were asked to complete a detailed weekly retrospective FFQ (i.e., over the last 5 years prior to RA onset). Healthy controls were required to complete a similar weekly retrospective FFQ, to gather information on their dietary intakes for the previous 5 years. FFQs were coded and analyzed. The patients and healthy controls with incomplete data from FFQs were excluded from the study.

The study was conducted in China between May 2012 and September 2013. A survey was designed following the guidelines established by the Chinese Rheumatology Association (CRA). The study was approved by the Medical Ethics Committee of Peking University People’s Hospital. Informed written consent forms were obtained from all study participants.

### Demographic data

Demographics and personal characteristics were gathered, including gender, age, marital status, education, occupation, personal income, height, and weight.

### Dietary assessment

Dietary assessment on the FFQ includes the frequency and the amount of dietary intake. The frequency of red meat, poultry, fish, edible viscera, vegetables, potatoes, mushrooms, beans, nuts, milk or yogurt, eggs, citrus, and other fruit except for citrus was measured with a response range from 1 to 8 (1 = less than once per month, 2 = 1–3 times per month, 3 = 1–2 times per week, 4 = 3–4 times per week, 5 = 5–6 times per week, 6 = 1 time per day, 7 = 2 times per day, and 8 = 3 or more times per day). The intake amount per serving of rice, flour meal, red meat, poultry, fish, edible viscera, vegetables, potatoes, mushrooms, beans, and nuts was measured with a response range from 1 to 4 (1 = 50 g or less, 2 = 50–100 g, 3 = 100–150 g, 4 = 150–200 g or more). The amount of eggs, citrus, and other fruit except for citrus was measured with a response range from 1 to 5 (1 = 1/2 or less, 2 = 1/2–2/3, 3 = 2/3–1, 4 = 1–2, 5 = 2–3 or more). The intake amount per serving of milk or yogurt was measured with a response range from 1 to 5 (1 = 50 ml or less, 2 = 50–75 ml, 3 = 75–100 ml, 4 = 100–200 ml, 5 = 200–300 ml or more). The total amount of dietary intake per month of a food item is equal to the intake amount per serving of that food item × the frequency of dietary intake.

### Statistical analyses

Differences in quantity of consumption between cases and controls were assessed using *t* test analyses. Multiple logistic regression analysis was applied to identify independent dietary risk factor(s) responsible for RA susceptibility. Odds ratios (ORs) with 95 % confidence intervals (CIs) were calculated to estimate the relative risk. *P* values less than 0.05 were considered significant.

## Results

### Demographic profile of RA patients

The study subjects were mainly recruited from two ethnic groups: Han (*n* = 913) and Hui (*n* = 18). Additionally, there were 37 subjects coming from other ethnic groups, including Man (*n* = 27), Mongol (*n* = 7), Yi (*n* = 1), and Zhuang (*n* = 1). The demographic characteristics of patients and controls are shown in Table 1.

**Table 1:** Characteristics of RA patients and healthy controls

	RA (*n* = 968)	HC (*n* = 1037)
Gender (%)		
Male	202 (20.9)	220 (21.2)
Female	766 (79.1)	817 (78.8)
Age (years), (mean ± SD)	52.1 ± 13.4	49.7 ± 15.2
Age group (years), (%)		
≤20	22 (1.3)	27 (2.6)
21–30	50 (5.2)	76 (7.3)
31–40	83 (8.7)	88 (8.5)
41–50	183 (19.1)	237 (22.9)
51–60	324 (33.8)	370 (35.7)
61–70	212 (22.1)	160 (15.4)
≥71	94 (9.8)	79 (7.6)
Ethnicity (%)		
Han	913 (94.3)	967 (93.2)
Hui	18 (1.9)	20 (1.9)
Others	37 (3.8)	50 (4.8)

*RA* rheumatoid arthritis, *HC* healthy controls

### Dietary intakes in RA patients and healthy controls

**Fig. 2:**
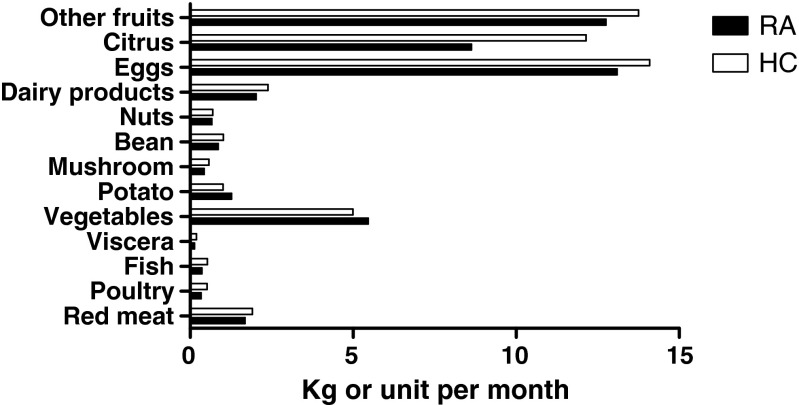
Dietary intakes of RA patients and healthy controls (*HC*). Compared with healthy controls, RA patients had higher amount of consumption in potatoes and lower amount of consumption in chicken, fish, mushrooms, beans, dairy products, citrus fruits, and organ meats. There were no significant differences in consumption of red meat between RA patients and healthy controls

The results of the dietary intakes of both groups are shown in Fig. 2 and Table 2. Compared with healthy controls, RA patients consumed lower amounts of chicken (*P* = 0.0001), fish (*P* = 0.0001), mushrooms (*P* = 0.0001), beans (*P* = 0.006), dairy products (*P* = 0.005), citrus fruits (*P* = 0.000), and organ meats (*P* = 0.018). There were no significant differences in consumption of red meat between RA patients and healthy controls.

**Table 2:** Differences in monthly dietary consumption between RA patients and healthy controls

	RA (*n* = 968)^a^	HC (*n* = 1037)^a^	*P*
Red meat (kg/month), mean ± SD	1.688 ± 1.621	1.911 ± 1.620	0.363
Poultry (kg/month), mean ± SD	0.339 ± 0.498	0.521 ± 0.890	0.000
Fish (kg/month), mean ± SD	0.361 ± 0.546	0.526 ± 1.029	0.000
Edible viscera (kg/month), mean ± SD	0.139 ± 0.224	0.195 ± 0.635	0.018
Potatoes (kg/month), mean ± SD	1.275 ± 1.884	1.015 ± 1.095	0.001
Vegetables (kg/month), mean ± SD	5.465 ± 4.368	4.997 ± 3.912	0.020
Mushrooms (kg/month), mean ± SD	0.427 ± 0.636	0.577 ± 0.989	0.000
Beans (kg/month), mean ± SD	0.866 ± 0.993	1.019 ± 1.301	0.006
Citrus fruits (unit/month), mean ± SD	8.630 ± 17.163	12.143 ± 20.726	0.000
Other fruits (unit/month), mean ± SD	12.753 ± 15.160	13.753 ± 20.824	0.268
Nuts (kg/month), mean ± SD	0.672 ± 1.420	0.696 ± 1.260	0.715
Dairy products (milk or yogurt) (L/month), mean ± SD	2.029 ± 2.697	2.388 ± 2.578	0.005
Eggs (unit/month) mean ± SD	13.093 ± 13.686	14.099 ± 14.566	0.143

*RA* rheumatoid arthritis, *HC* healthy controls

^**a**^Entries may not add up to 968 or 1037 because of the responses obtained

### Comparison of dietary intake between Han and Hui populations in both RA and HC groups

We compared the dietary consumption between the Han and Hui populations in the RA and HC cohorts, respectively. As shown in Table 3, except for dairy products, there were no significant differences in dietary consumption between Han and Hui groups.

**Table 3:** Differences in monthly dietary consumption between Han patients and Hui patients

	HC			RA		
	Han (*n* = 967)^a^	Hui (*n* = 20)^a^	*P*	Han (*n* = 913)^a^	Hui (*n* = 18)^a^	*P*
Red meat (kg/month), mean ± SD	1.838 ± 1.781	2.733 ± 2.309	0.136	1.652 ± 1.924	1.144 ± 1.095	0.431
Poultry (kg/month), mean ± SD	0.495 ± 0.626	1.565 ± 4.265	0.317	0.345 ± 0.516	0.306 ± 0.246	0.765
Fish (kg/month), mean ± SD	0.514 ± 0.862	0.484 ± 0.345	0.893	0.373 ± 0.563	0.327 ± 0.256	0.750
Edible viscera (kg/month), mean ± SD	0.172 ± 0.414	0.181 ± 0.206	0.940	0.135 ± 0.210	0.181 ± 0.383	0.394
Potatoes (kg/month), mean ± SD	1.023 ± 1.104	0.632 ± 0.501	0.146	1.269 ± 1.823	0.683 ± 0.662	0.174
Vegetables (kg/month), mean ± SD	5.025 ± 3.901	5.033 ± 3.458	0.993	5.538 ± 4.361	5.182 ± 4.345	0.739
Mushrooms (kg/month), mean ± SD	0.586 ± 1.018	0.553 ± 0.389	0.902	0.430 ± 0.651	0.462 ± 0.479	0.844
Beans (kg/month), mean ± SD	1.026 ± 1.319	0.829 ± 0.731	0.541	0.883 ± 1.012	0.681 ± 0.725	0.429
Citrus fruits (unit/month), mean ± SD	11.767 ± 19.992	20.359 ± 29.988	0.256	8.709 ± 17.639	6.350 ± 5.475	0.582
Other fruits (unit/month), mean ± SD	13.413 ± 19.391	14.421 ± 11.191	0.831	12.778 ± 15.438	10.188 ± 9.941	0.504
Nuts (kg/month), mean ± SD	0.691 ± 1.271	0.935 ± 1.199	0.433	0.654 ± 1.378	1.278 ± 3.143	0.412
Dairy products (milk or yogurt) (L/month), mean ± SD	2.366 ± 2.581	2.385 ± 3.198	0.976	1.984 ± 2.550	3.491 ± 2.976	0.020*
Eggs (unit/month), mean ± SD	14.115 ± 14.276	19.429 ± 28.746	0.472	13.352 ± 13.956	10.006 ± 13.4103	0.374

*RA* rheumatoid arthritis, *HC* healthy controls

^a^Entries may not add up to 967 or 20 or 913 or 18 because of the responses obtained

*p < 0.05

### Comparison of dietary intakes between Han and non-Han in both RA and HC groups

We compared the dietary consumption between the Han and non-Han populations in both RA and HC groups. Han people with RA intake more fish and less dairy products than non-Han people with RA. There were no other significant differences in dietary consumption between the Han and non-Han groups (Table 4).

**Table 4:** Differences in monthly dietary consumption between Han patients and Non-Han patients

	HC			RA		
	Han (*n* = 967)^a^	Non-Han (*n* = 70)^a^	*P*	Han (*n* = 913)^a^	Non-Han (*n* = 55)^a^	*P*
Red meat (kg/month) mean ± SD	1.838 ± 1.781	3.400 ± 4.517	0.139	1.652 ± 1.924	1.456 ± 1.522	0.608
Poultry (kg/month) mean ± SD	0.495 ± 0.626	0.878 ± 2.843	0.406	0.345 ± 0.516	0.279 ± 0 .215	0.420
Fish (kg/month) mean ± SD	0.514 ± 0.862	0.849 ± 2.921	0.490	0.373 ± 0.563	0.251 ± 0.247	0.009*
Edible viscera (kg/month) mean ± SD	0.172 ± 0.414	0.614 ± 2.330	0.285	0.135 ± 0.210	0.231 ± 0.432	0.197
Potatoes (kg/month) mean ± SD	1.023 ± 1.104	0.826 ± 0 .699	0.254	1.269 ± 1.823	1.341 ± 2.763	0.809
Vegetables (kg/month) mean ± SD	5.025 ± 3.901	4.906 ± 3.978	0.839	5.538 ± 4.361	4.664 ± 4.134	0.222
Mushrooms (kg/month) mean ± SD	0.586 ± 1.018	0.473 ± 0 .405	0.491	0.430 ± 0.651	0.422 ± 0.459	0.936
Beans (kg/month) mean ± SD	1.026 ± 1.319	0.884 ± 1.077	0.519	0.883 ± 1.012	0.722 ± 0.718	0.328
Citrus fruits (unit/month) mean ± SD	11.767 ± 19.992	17.317 ± 23.719	0.079	8.709 ± 17.639	9.277 ± 11.397	0.841
Other fruits (unit/month) mean ± SD	13.413 ± 19.391	16.093 ± 13.838	0.400	12.778 ± 15.438	12.492 ± 10.974	0.909
Nuts (kg/month) mean ± SD	0.691 ± 1.271	0.682 ± 0.945	0.964	0.654 ± 1.378	0.955 ± 2.140	0.180
Dairy products (milk or yogurt) (L/month) mean ± SD	2.366 ± 2.581	2.495 ± 2.540	0.747	1.984 ± 2.550	3.557 ± 4.677	0.000*
Eggs (unit/month) mean ± SD	14.115 ± 14.276	13.752 ± 19.794	0.878	13.352 ± 13.956	11.043 ± 10.580	0.309

*RA* rheumatoid arthritis, *HC*, healthy controls

^a^Entries may not add up to 967 or 70 or 913 or 55 because of the responses obtained

*p < 0.05

### Dietary intake as independent risk factor for RA development

The multivariate analyses revealed that females and individuals older than 50 years of age were more susceptible to RA (Table 5). Consumption of several dietary items were independent factors for higher risk of RA development, including potatoes (OR = 1.160; 95 % CI = 1.035–1.300, *P* = 0.011) and fruits except for citrus fruits (OR = 1.013; 95 % CI = 1.003–1.023, *P* = 0.013). In contrast, some dietary items were found to have protective effects on RA, including mushrooms (OR = 0.669; 95 % CI = 0.518–0.864, *P* = 0.002), dairy products (OR = 0.921; 95 % CI = 0.867–0.977, *P* = 0.006), and citrus fruits (OR = 0.990; 95 % CI = 0.981–0.999, *P* = 0.040). Consumption of red meats, fruits, and vegetables was not found to have any effect on RA risk. The analyses revealed that higher intakes of carbohydrates and lower intakes of dairy products, mushrooms, and citrus fruits may associate with RA development. There was a trend for high intakes of fish (OR = 0.864; 95%CI = 0.647–1.156, *P* = 0.325) and beans (OR = 0.938; 95%CI = 0.811–1.086, *P* = 0.391) to have protective effects against RA, though these did not reach statistical significance. Additionally, no significant associations were observed between the consumption of red meat and RA risk.

**Table 5:** Multiple logistic regression of factors associated with RA (*n* = 968)

	Β	OR (95 % CI)	*P*
Gender (male, reference group)	0.875	2.398 (1.722–3.340)	0.000
Age group (≤20, reference group)			
21–30	−0.476	0.621 (0.228–1.689)	0.351
31–40	0.154	1.167 (0.425–3.205)	0.765
41–50	0.506	1.658 (0.627–4.390)	0.308
51–60	1.227	3.411 (1.305–8.914)	0.012
61–70	1.712	5.543 (2.033–15.112)	0.001
≥71	1.336	3.803 (1.305–11.082)	0.014
Ethnicity (Han, reference group)			
Hui	0.719	2.051 (0.342–12.289)	0.431
Others	0.250	1.284 (0.426–3.872)	0.657
Red meat (kg/month)	0.046	1.047 (0.945–1.160)	0.382
Poultry (kg/month)	−0.119	0.887 (0.672–1.171)	0.399
Fish (kg/month)	−0.146	0.864 (0.647–1.156)	0.325
Edible viscera (kg/month)	0.138	1.148 (0.716–1.842)	0.566
Potato (kg/month)	0.148	1.160 (1.035–1.300)	0.011*
Vegetables (kg/month)	0.029	1.030 (0.991–1.070)	0.135
Mushroom (kg/month)	−0.402	0.669 (0.518–0.864)	0.002*
Bean (kg/month)	−0.064	0.938 (0.811–1.086)	0.391
Citrus (unit/month),	−0.010	0.990 (0.981–0.999)	0.040*
Other fruits except for citrus (unit/month)	0.013	1.013 (1.003–1.023)	0.013*
Dairy products (L/month)	−0.083	0.921 (0.867–0.977)	0.006*
Eggs (unit/month)	−0.004	0.996 (0.984–1.008)	0.502
BMI	0.011	1.011 (0.975–1.048)	0.559

*RA* rheumatoid arthritis

*p < 0.05

## Discussion

The etiology of RA remains an area of considerable interest. RA is triggered by genetic factors; however, environmental factors may also play a role in the pathogenesis of RA. Dietary and lifestyle factors (e.g., smoking) [2, 3] contribute to RA. Smoking is so far the most well-established environmental risk factor for development of RA [2, 3]. Additionally, infectious agents such as viruses, bacteria, and fungi have long been suspected risk factors for RA, but there is no conclusive evidence to support the hypothesis [14]. Influences of other environmental factors on RA, such as hormones, have also been suggested. Hormones are believed to influence RA based on the fact that females are more prone to develop RA than are men, with a peak onset at 50–60 years of age [1, 15].

Salminen et al. [15] reported that 33–75 % patients believe that food plays an important role in their symptom severity and approximately 50 % will have tried dietary manipulation in an attempt to improve their symptoms. Diets rich in fiber, omega-3 fatty acids, and antioxidants, and low in red meat have been reported associated with decreased RA risks [16–18]. Comparing with the survey by others [16–18], Chinese diets are characterized by higher levels of vegetables and lower intakes of red meat consumption and the average meat consumption is much lower than Western diets. In this study, there were no significant differences in red meat intakes between RA patients and healthy controls, as reported in other studies [19, 20]. Whether the association between red meat consumption and inflammatiory arthritis remains unclear, meat consumption might affect the gut microbiota or contribute to high energy intakes, which provide some explanation for the association.

The results of this study revealed that high intakes of carbohydrates (e.g., potatoes) might be associated with increased RA risks. High carbohydrate and lower fiber intakes lead to excess energy intake. With excess energy intake and reduced energy expenditure, body weight and adiposity increase. Although there was no signifcant difference in body mass index (BMI) between RA patients and HCs in present work, other studies have reported that BMI is positively correlated with chronic inflammatory disorders [21]. It is recognized that adipocytes release the proinflammatory cytokines TNF, IL-1β and IL-6; thus, adipose tissue is metabolically active and contribute to sysytemic inflammatory responses [3, 21].

Studies have reported that ROS are involved in the pathogenesis of RA. Antioxidant-rich diets have beneficial effects on several diseases. However, in this study, high intakes of fruits or vegetables had no significant effects. Interestingly, citrus fruits have a protective role in the pathogenesis of RA. Citrus fruits are rich in hesperidin. Kawaguchi [22] reported that the consumption of citrus flavanone and naringin suppressed the inflammatory responses in collagen-induced arthritis in mice, possibly by decreasing tumor necrosis factor-α (TNF-α) levels. In an 8-week, placebo-controlled, randomized, double-blind clinical trial, Oben et al. reported that citrus extracts improved knee joint pain and flexibility and reduced C-reactive protein levels [23]. In addition to hesperidin and naringin, citrus fruits are rich in vitamin C, which has protective roles according to some studies and no clinical benefits according to other studies [11].

Mushroom consumption was significantly lower in RA patients than in healthy controls. Mushrooms have food and pharmaceutical applications due to the presence of β-glucans, polysaccharopeptides, and polysaccharide-protein conjugates, which have immunomodulatory and antitumor activities. These compounds have demonstrated mitogenicity and activation of immune effector cells, such as lymphocytes, macrophages, and natural killer cells, resulting in the production of cytokines, including interleukins, TNF-α, and interferon gamma [24]. Yu et al. reported that mushrooms regulate immunity in vitro [25]. A number of mushroom components have been shown to modulate immunity and therefore might play a role in preventing RA.

In this study, there were no significant benefits from fish consumption; however, fish contains omega-3 fatty acids, which have protective roles. More than 20 randomized controlled trials have reported that omega-3 fatty acids have modest benefits on RA treatment. Additionally, symptoms got worse in RA patients who discontinued the fish oil supplements [16, 26, 27].

Dairy products, such as milk and yogurt, had protective roles against RA in this study. Yogurt contains probiotics, which maintain a healthy gut microbiota. A metagenomic approach using Illumina sequencing of pooled milk samples revealed that the genera and types of bacteria in milk may affect bacterial establishment and stability in this food matrix [28]. Supplementation with *Lactobacillus casei* improves the inflammatory status of patients with RA [29]. A 3-month, double-blind, placebo-controlled study reported considerable improvements in RA patients following supplementation with *Lactobacillus rhamnosus* and *Lactobacillus reuteri* [30]. Additionally, the administration of probiotics in different animal models improved inflammatory bowel disease, atopic dermatitis, and RA, probably as a result of enrichment of CD4^+^Foxp3^+^ Tregs in inflamed body areas [31]. Therefore, probiotics are recommended to patients with RA.

In conclusion, dietary factors contribute to the development of RA. Dietary modification might reduce RA risk and prevent disease progression. There is a need for large-scale prospective, placebo-controlled studies to assess the effects of multiple dietary compounds on RA.
